# Association between remote resistance exercises programs delivered by a smartphone application and skeletal muscle mass among elderly patients with type 2 diabetes– a retrospective real-world study

**DOI:** 10.3389/fendo.2024.1407408

**Published:** 2024-06-11

**Authors:** Jing Yang, Hongyu Tan, Haoyan Yu, Jingshuo Li, Yang Cui, Yuanjian Lu, Xin Liu, Qimin Chen, Daan Zhou

**Affiliations:** ^1^Department of Rehabilitation, The Third Affiliated Hospital of Jinzhou Medical University, Liaoning, China; ^2^Postgraduate Training Basement, Jinzhou Medical University, Liaoning, China; ^3^Department of Radiology, Shanghai Sixth People’s Hospital Affiliated to Shanghai Jiao Tong University School of Medicine, Shanghai, China; ^4^Department of Traditional Chinese Medicine, The First Affiliated Hospital of Guangxi Medical University, Guangxi, China; ^5^Department of Orthopedics, The Third Affiliated Hospital of Jinzhou Medical University, Liaoning, China

**Keywords:** resistance exercises, smart phone application, skeletal muscle mass, diabetes mellitus, remote rehabilitation, elderly

## Abstract

**Objective:**

We aimed to explore the relationship between remote resistance exercise programs delivered via a smartphone application and skeletal muscle mass among elderly patients with type 2 diabetes, utilizing real-world data.

**Methods:**

The resistance exercises were provided through Joymotion®, a web-based telerehabilitation smartphone application (Shanghai Medmotion Medical Management Co., Ltd). The primary outcome was the changes in skeletal muscle index (SMI) before and after the remote resistance exercises programs. The secondary outcomes were changes in skeletal muscle cross-sectional area (SMA), skeletal muscle radiodensity (SMD) and intermuscular adipose tissue (IMAT).

**Results:**

A total of 101 elderly patients with type 2 diabetes were analyzed. The participants had an average age of 72.9 ± 6.11 years for males and 74.4 ± 4.39 years for females. The pre- and post-intervention SMI mean (± SE) was 31.64 ± 4.14 vs. 33.25 ± 4.22 cm^2^/m^2^ in male, and 22.72 ± 3.24 vs. 24.28 ± 3.60 cm^2^/m^2^ in female respectively (all P < 0.001). Similarly, a statistically significant improvement in SMA, IMAT, and SMD for both male and female groups were also observed respectively (P < 0.001). Multiple linear regression models showed potential confounding factors of baseline hemoglobin A1c and duration of diabetes with changes in SMI in male, while hemoglobin A1c and high density lipoprotein cholesterol with changes in SMI in female.

**Conclusion:**

Remote resistance exercises programs delivered by a smartphone application were feasible and effective in helping elderly patients with type 2 diabetes to improve their skeletal muscle mass.

## Introduction

Elderly patients with type 2 diabetes (T2D) are a distinct and rapidly expanding group, confronting a wide array of health challenges, including a heightened risk of developing sarcopenia (1). Sarcopenia, defined by the loss of muscle mass, strength, and function, significantly diminishes quality of life, functional independence, and healthcare outcomes in this demographic (2). The relationship between T2D and sarcopenia in elderly adults is intricate and reciprocal. T2D propels the aging process via several mechanisms, such as chronic inflammation, oxidative stress, and endothelial dysfunction, all of which contribute to the development and progression of sarcopenia. In turn, the presence of sarcopenia can exacerbate diabetes management, complicating glycemic control, increasing the likelihood of hypoglycemia, and elevating the risk of hospital admissions, falls and fractures, and even death (3, 4). Additionally, there is an increasing body of evidence indicating that both sarcopenia and T2D independently and together play a role in cognitive deterioration, elevating the risk of developing dementia, including Alzheimer’s disease (5–7).

Resistance exercise, also known as strength training, is crucial in managing T2D and addressing sarcopenia, especially among the elderly population (8–10). This physical activity entails exercises that force muscles to contract against an external force, aiming for improvements in strength, tone, mass, and/or endurance. The advent of remote resistance exercise programs available through smartphone applications marks a significant progression in healthcare technology (11, 12). These innovations provide accessible, personalized, and scalable exercise solutions, featuring real-time feedback and instructions, some of which can integrate with wearable technology. While several clinical trials have shed light on the effectiveness of such interventions in clinical scenarios (13–15), home-based real-world evidence, particularly within the Chinese population, remains scarce. Our objective is to explore the relationship between remote resistance exercise programs delivered via a smartphone application and skeletal muscle mass among elderly patients with T2D, utilizing real-world data.

## Methods

### Study design and participants

**Figure 1:**
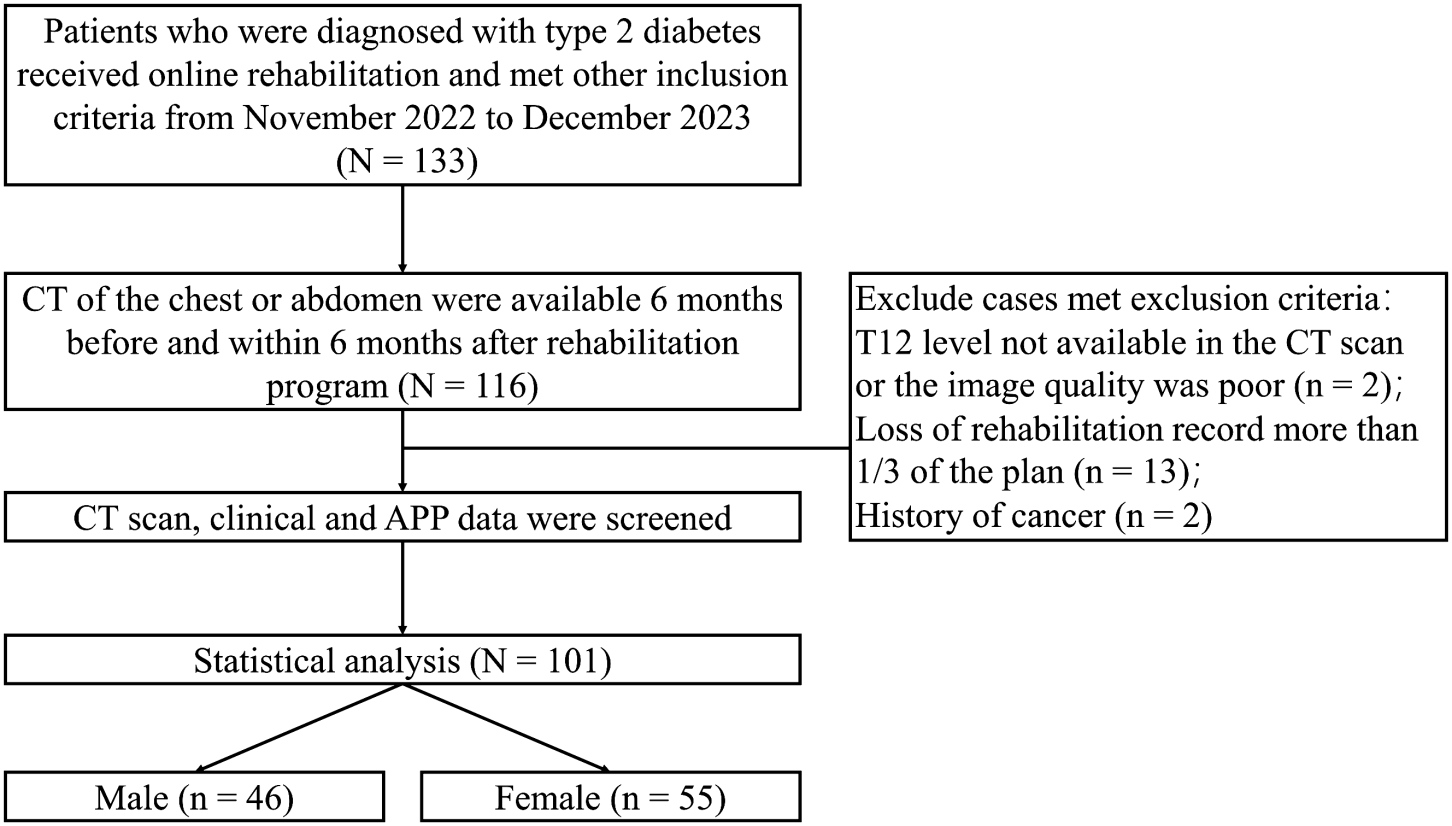
Participant flow diagram.

The Department of Rehabilitation at the Third Affiliated Hospital of Jinzhou Medical University offers a comprehensive array of services, including a rehabilitation medicine clinic and specialized clinics. It is recognized as the most expansive and technologically advanced rehabilitation facility among the large comprehensive hospitals in western Liaoning, boasting expertise in several key areas such as exercise prescriptions, neurological rehabilitation, trauma rehabilitation, and traditional medicine rehabilitation. We extracted data from the electronic health records (EHRs) from the Third Affiliated Hospital of Jinzhou Medical University. Eligible patients were (1) aged 65 years or older; (2) confirmed diagnosis of T2D; (3) using remote resistance exercises programs for at least 3 months; (3) available data on clinical characteristics and medical history. Exclusion criteria included: (1) patients without available images in the CT scan or with poor image quality; (2) patients with loss of rehabilitation record; (3) without complete clinical data (see **Figure 1** in detail). The analytic plan was approved by the Institutional Review Board of the Third Affiliated Hospital of Jinzhou Medical University (JYDSY-KXYJ-IEC-2021–009). Our study did not require obtaining informed consent from individual participants as it utilized retrospective anonymized data extracted from EHRs.

### Data source and measurements

Using the Chinese government-issued unique personal identification numbers, we developed an electronic database. This database compiled information such as date of birth, sex, age at diabetes diagnosis, smoking status, and medication use, including antihypertensive, glucose-lowering, lipid-lowering, and antiplatelet or anticoagulant drugs. Data was gathered via a standardized form for collecting electronic inpatient and outpatient medical record information. Additionally, at every inpatient admission or outpatient visit, standardized methods were used to measure patients’ height, weight, blood pressure, plasma glucose levels, C-peptide, total cholesterol (TC), triglycerides (TG), high-density lipoprotein (HDL) cholesterol, low-density lipoprotein (LDL) cholesterol, hemoglobin A1c (HbA1c), serum creatinine, and C-reactive protein. The body mass index (BMI) was calculated using the formula weight in kilograms (kg) divided by the square of height in meters (m²). The estimated glomerular filtration rate (eGFR) was determined using the formula from the Chronic Kidney Disease Epidemiology Collaboration (CKD-EPI) (16). Comorbidities and history of diseases were coded using the International Classification of Diseases 10th version (ICD-10).

### Remote resistance exercises programs delivered by smartphone application

The resistance exercises were provided through Joymotion®, a web-based telerehabilitation smartphone application (Shanghai Medmotion Medical Management Co., Ltd) (17). Joymotion®; is a remote rehabilitation and exercise prescription monitoring device designed for treating musculoskeletal diseases. It integrates wearable sensor hardware with an app that features a library of standardized exercise prescriptions based on evidence-based practices. These can be tailored by doctors to create personalized treatment plans. The app guides patients through exercises with video and written instructions, monitors performance in real-time, and offers interactive communication with online physiotherapists. It also pushes educational content tailored to patients’ conditions, enhancing understanding and engagement in their rehabilitation. Key features include the ability to facilitate easy follow-ups for doctors and automatically push tele-rehabilitation as “daily tasks” and targeted educational videos, improving patient education and compliance. Through real-time tracking, messaging, and video consultations, Joymotion® facilitates continuous personalized care and supports comprehensive disease management, even after hospital discharge. Within the app, immediate feedback on the completion of exercises within a session is recorded, determining success based on whether the patient fully viewed or skipped the instructional videos.

In this study, a 12-week progressive elastic band resistance training program was delivered via the Joymotion®. Participants engaged in a structured series of resistance exercises employing elastic bands (Thera-Band®, The Hygenic Corporation, Akron, OH, USA). These bands, differentiated by color (yellow, red, green, blue, black, or silver), provided varying levels of resistance, with about 20% increase in resistance intensity. The training protocol was meticulously designed to encompass all major muscle groups, incrementally elevating both the volume and intensity of the exercises. Training intensities were meticulously tailored to the individual’s clinical profile and historical health data, ensuring a bespoke rehabilitation process. Adherence to the resistance training guidelines established by the American College of Sports Medicine for elderly adults was ensured (18).

Participant compliance and safety were paramount; thus, participants were instructed to report the perceived exertion and any difficulties encountered during each session through the application. Weekly virtual consultations with the physical therapist were mandated to evaluate training load, ascertain the presence of any adverse reactions, and adjust training intensity and exercises progressively optimizing the intervention protocol based on these evaluations. A series of elastic bands with varying levels of resistance were provided, allowing for progression based on participants’ training capacity. In instances where participants were unable to tolerate the increased intensity of the next resistance level, the resistance intensity of the elastic band was maintained at the previous level for an additional session. However, in such cases, the number of repetitions per set might be increased to continue challenging the participants and promoting strength development. This maintenance continued until participants could successfully achieve a 20% increase in resistance intensity, signaling readiness to progress to the next color-coded elastic band. The program stipulated a thrice-weekly engagement over 12 weeks, culminating in 36 sessions. Each session was structured to last 50 minutes, comprising a 5-minute general warm-up, 40 minutes of targeted elastic band resistance exercises, and a concluding 5-minute cool-down and stretching phase. The exercise component was designed to target specific muscle groups—including the lower limbs, core, arms, and shoulders—through 1 or 2 exercise variations. For each exercise, participants were instructed to perform 2–3 sets of 8–12 repetitions, focusing on controlled concentric and eccentric muscle contractions across the full movement range, facilitated by the resistance offered by the elastic bands.

### Evaluation of skeletal muscle mass

We used computed tomography (CT) images at the thoracic 12 (T12) vertebral level to assess the muscle area and quality. This method is commonly used in many other studies for assessing sarcopenia or the loss of skeletal muscle mass and function (19–21). Since the COVID-19 pandemic in 2019, chest CT scanning is routine at every clinical visit and the reports and images were stored in the EHR systems. The T12 vertebra is identified on the CT scan by locating the last rib’s attachment point, as T12 is the last thoracic vertebra to which ribs attach. We used SliceOmatic 6 (TomoVision, Magog, Canada) to analyze the images. The skeletal muscle cross-sectional area (SMA) at the T12 level is delineated manually. The muscles analyzed include the paraspinal muscles (erector spinae and multifidus), and intercostal muscles. The software calculated the total cross-sectional area of skeletal muscle in square centimeters (cm²) and we further adjusted these measurements for height to derive a skeletal muscle index (SMI). Skeletal muscle radiodensity (SMD) and intermuscular adipose tissue (IMAT) were determined using the images at T12 level based on the Hounsfield Unit (HU) values. The SMD and IMAT were reported as the mean HU value within the erector spinae muscle area.

### Outcomes

The primary outcome was the changes in SMI before and after the remote resistance exercises programs. The secondary outcomes included the changes in SMA, SMD and IMAT.

### Statistical analysis

For the primary outcome measure of SMI, we determined that a sample size of 44 patients in each gender would grant the study 90% statistical power, with a two-sided alpha level of 0.05, in a two-sided paired t-test, to detect a treatment difference of 2 cm^2^/m^2^ in male and 1.5 cm^2^/m^2^ in female, assuming a standard deviation of 4 in male and 3 in female. G*power version 3.1 (Heinrich-Heine-Universität Düsseldorf Universitätsstr, Germany) was employed in the calculation (22). To accommodate potential exclusion, we increased the target enrollment to 133 patients.

Data with a normal distribution were described using the mean and standard deviation, while skewed data were presented as the median alongside interquartile ranges. Frequencies and percentages were used to describe categorical variables. To compare between groups, Student’s t-tests were applied to normally distributed data, the Wilcoxon rank-sum test was used for skewed data, and the χ2 test was utilized for categorical variables. Baseline variables that were considered clinically relevant or that showed a univariate relationship with outcome were entered into multiple regression model (**Table 1**). Variables for inclusion were carefully chosen, given the number of events available, to ensure parsimony of the final model. Stepwise multiple linear regression models (*lmtest, caret* in R) were used to estimate the confounding factors associated with the outcomes (**Table 2**). The primary analysis was performed according to sexes separately. The model employed the Breusch-Pagan test to assess the homoscedasticity of the residuals for the validity of Ordinary Least Squares (OLS) regression. A P-value of less than 0.05 was considered of statistical significance. The R software version 4.3.3 (R Foundation for Statistical Computing, Vienna, Austria) was utilized to carry out all the statistical analysis.

**Table 1:** Univariable regression analysis to explore the associations of baseline characteristics with ΔSMI.

	Male	R^2^	P- value	Female	R^2^	P- value
	β			β		
Age	-0.389	0.152	0.007	0.006	0.019	0.967
BMI	0.138	-0.003	0.360	0.057	-0.016	0.681
HbA1c	0.922	0.847	<0.001	0.954	0.908	<0.001
LDL	0.031	-0.022	0.837	0.168	0.010	0.220
HDL	0.256	0.044	0.086	-0.321	0.086	0.017
TG	-0.321	0.022	0.161	-0.321	0.012	0.203
eGFR	0.164	0.005	0.276	-0.132	-0.001	0.336
Insulin	-0.145	-0.001	0.336	0.417	0.159	0.002
Oral glucose lowering drugs	0.095	-0.014	0.530	0.225	0.033	0.098
Anti-hypertension drugs	-0.205	0.020	0.171	0.144	0.002	0.294
Statins	-0.029	-0.022	0.847	0.135	0.000	0.325
Aspirin	-0.100	-0.012	0.507	-0.025	-0.018	0.858
Duration	0.800	0.631	<0.001	-0.063	-0.015	0.648

R^2^, adjusted R^2^.

**Table 2:** Multiple linear regression analysis for ΔSMI.

	Variable	Co-efficient	95% CI	P- value
Male	HbA1c	0.208	0.164, 0.251	<0.001
	Duration	0.030	0.014, 0.047	<0.001
Female	HbA1c	0.349	0.320, 0.378	<0.001
	HDL	-0.004	-0.006, -0.002	<0.001

## Results

### Baseline characteristics

From November 2022 to December 2023, 133 patients were screened for eligibility, and 101 were analyzed, with 46 male (45.5%) and 55 female (54.5%) patients (**Figure 1**). The demographic and clinical characteristics of the patients at baseline are shown in **Table 3**. The participants had an average age of 72.9 ± 6.11 years for males and 74.4 ± 4.39 years for females (P < 0.05). The mean diastolic blood pressure (DBP) was 72.3 ± 5.62 mmHg for males and 70.8 ± 5.60 mmHg for females. Systolic blood pressure (SBP) was 132 ± 9.08 mmHg for males and 136 ± 10.8 mmHg for females. BMI was balanced across genders, with males at 28.0 ± 3.76 kg/m^2^ and females at 28.2 ± 4.43 kg/m^2^. Medication usage varied across the cohort, with higher percentages using insulin (male 50%, female 47.2%) in male and metformin (male 54.3%, female 70.9%) in female. Anti-hypertensive drug use was prevalent, with over 89% of each gender using these medications. Statin use was also high, with 76.1% of males and 78.2% of females on these cholesterol-lowering drugs. Smoking status was reported by 34.8% of males and 27.3% of females (P < 0.05).

**Table 3:** Baseline characteristics of the patients.

	Male (n = 46)	Female (n = 55)
Age, yrs	72.9 (6.11)	74.4 (4.39)^*^
Height, cm	170 (6.31)	154 (6.33)
Weight, kg	80.8 (12.8)	67.4 (11.5)
DBP, mmHg	72.3 (5.62)	70.8 (5.60)
SBP, mmHg	132 (9.08)	136 (10.8)^*^
BMI, kg/m^2^	28.0 (3.76)	28.2 (4.43)
LDL, mmol/L	2.32 (0.64)	2.47 (0.80)
HbA1c, %	7.28 (1.38)	7.15 (1.02)
HDL, mmol/L	1.08 (0.29)	1.33 (0.35)
Tg, mmol/L	1.50 (0.73)	1.34 (0.52)
eGFR, mL/min/1.73m^2^	70.4 (27.2)	85.4 (28.7)^*^
Duration of diabetes, yrs	10.8 (3.59)	11.0 (4.26)
insulin, n(%)	23 (50.0)	26 (47.2)^*^
SUs, n(%)	13 (28.3)	23 (41.8)
metformin, n(%)	25 (54.3)	39 (70.9)^*^
DPP 4, n(%)	11 (23.9)	9 (16.4)
AGI, n(%)	0 (0.00)	1 (1.82)
SGLT 2 inhibitors, n(%)	1 (2.17)	0 (0.00)
GLP 1, n(%)	3 (6.52)	4 (7.27)
TZD, n(%)	3 (6.52)	1 (1.82)
Oral glucose lowering drugs, n(%)	40 (87.0)	49 (89.1)
Beta Blockers, n(%)	30 (65.2)	33 (60.0)
Calcium Channel Blockers, n(%)	24 (52.2)	34 (61.8)^*^
ACE Inhibitors, n(%)	27 (58.7)	31 (56.4)
ARBs, n(%)	14 (30.4)	17 (30.9)
Anti-hypertension drugs, n(%)	41 (89.1)	51 (92.7)
Statins, n(%)	35 (76.1)	43 (78.2)
Aspirin, n(%)	13 (28.3)	14 (25.5)^*^
Smoking, n(%)	16(34.8)	15(27.3)^*^

Data were mean (SD) or number (percentage).

^*^P < 0.05.

### The primary and secondary outcomes

**Figure 2:**
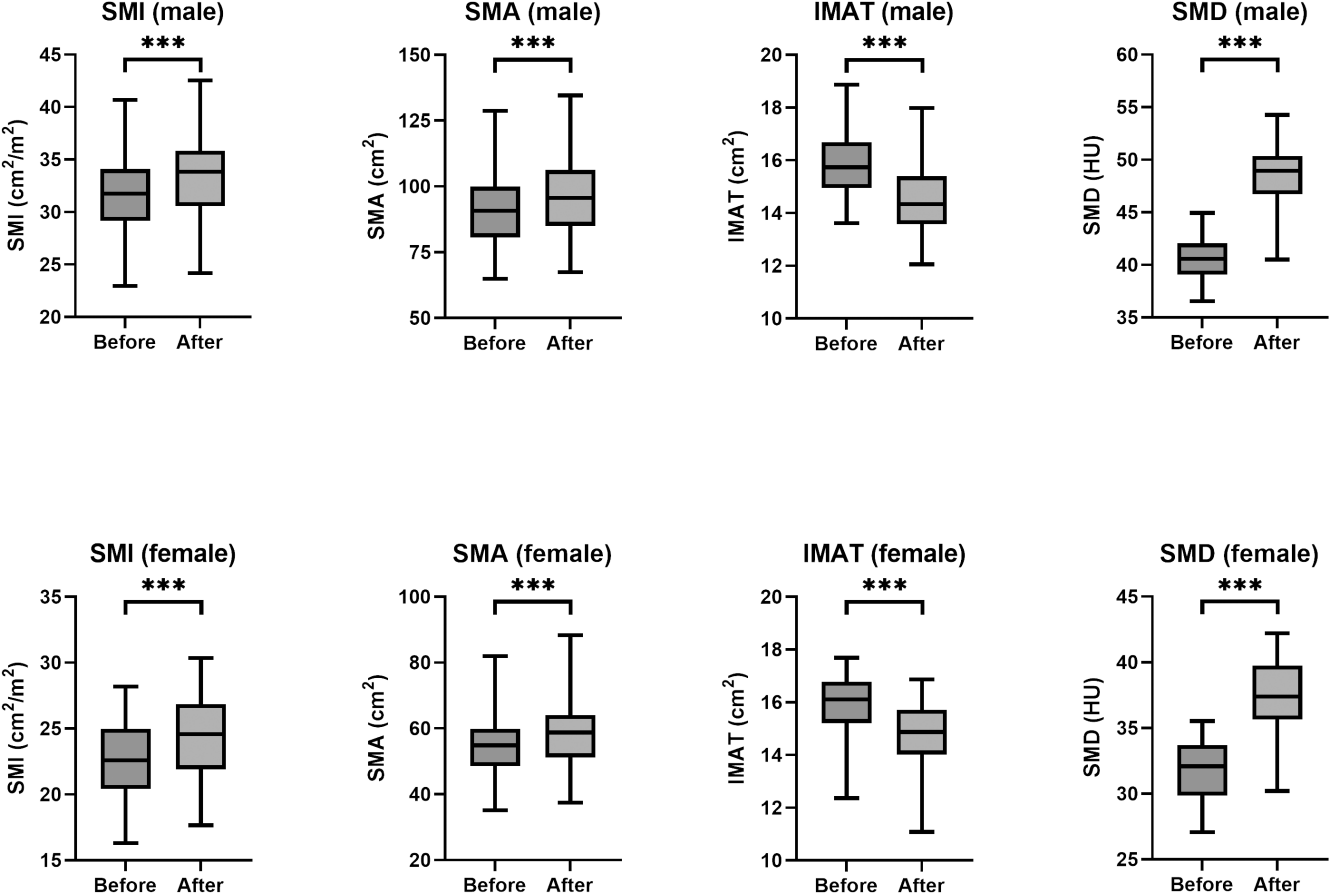
Box plots of SMI, SMA, IMAT, and SMD values before and after training by sex. HU, Hounsfield unit. (*** *P* < 0.001).

The primary outcome assessed in this study was the change in SMI following participation in a remote resistance exercise program (**Figure 2**). A statistically significant improvement in SMI for both male and female groups were observed after the program when compared to the SMI at baseline (P < 0.001). The pre- and post-intervention mean SMI was 31.64 ± 4.14 vs. 33.25 ± 4.22 cm^2^/m^2^ in male, and 22.72 ± 3.24 vs. 24.28 ± 3.60 cm^2^/m^2^ in female respectively (all P < 0.001). The secondary outcomes were the changes in SMA, IMAT, and SMD (**Figure 2**). Similarly, a statistically significant improvement in SMA, IMAT, and SMD for both male and female groups was also found respectively (P < 0.001).

### Multiple linear regression models

We further explored the correlations between the changes in SMI before and after the resistance training program (ΔSMI), and various baseline characteristics of patients were analyzed separately in univariate models (**Table 1**), then a forward stepwise approach used to inform the final variables for inclusion into the multiple regression model (**Table 2**). The analysis was conducted separately for male and female groups to identify variables significantly associated with ΔSMI within each gender. In the male group, HbA1c and duration of diabetes showed significant positive association with ΔSMI (HbA1c, coefficient 0.208 (0.164, 0.251), P < 0.001; duration of diabetes, coefficient 0.030 (0.014, 0.047), P < 0.001). In the female group, HbA1c was also found to have a significant positive correlation with ΔSMI (coefficient 0.349 (0.320, 0.378), P <0.001). HDL cholesterol levels were negatively associated with ΔSMI (coefficient -0.004 (-0.006, -0.002), P <0.001).

## Discussion

In this study, we demonstrated the significant improvements in SMI, SMA, IMAT, and SMD for both males and females by a remote resistance exercise program. Multiple linear regression analysis highlighted significant correlations between post-interventional SMI and various factors. These findings underscore the effectiveness of resistance training in enhancing muscle metrics among older adults, along with the complex interplay of metabolic health factors in modulating these outcomes.

The effectiveness of resistance exercises in addressing sarcopenia, particularly among elderly patients with T2D, has been substantiated by some clinical trials and studies. One prospective study (23) aimed to assess the impact of combined strength and aerobic exercises on frailty levels in elderly diabetic patients over 70 years of age with relatively high functional and cognitive capacities. The study demonstrated that a 6-month regimen of strength exercises with elastic bands and aerobic activities can effectively reduce frailty prevalence among elderly diabetic patients. Another randomized clinical trial (24) assessed the impact of high-protein diets both with and without resistance exercise training on weight loss, body composition, and cardiovascular disease risk factors over a 16-week period. The study found significant group effects for body weight, fat mass, and waist circumference, with the most substantial reductions observed in the low fact hypocaloric diet + resistance exercise group. Specifically, this group experienced the largest decrease in body weight (-13.8 ± 6.0 kg), fat mass (-11.1 ± 3.7 kg), and waist circumference (-13.7 ± 4.6 cm). Bouchi et al (25) also assessed the impact of combining resistance training, with the sodium-glucose cotransporter 2 inhibitor dapagliflozin on body composition and metabolic health in type 2 diabetes patients over a 24-week period of intervention. While this trial revealed no significant difference between the two groups in terms of changes in fat-free mass and SMI, a notable reduction in trunk fat mass was observed in the combination group. Our study showed notable improvements in the SMI following participation in remote resistance exercise programs, alongside a reduction in IMAT. These findings indicate the effectiveness of such a remote program, though further research is required to substantiate these results.

Certain gaps between clinical trials and real-world evidence may limit the interpretation of the findings above. Differences in population diversity, intervention adherence, data collection and outcomes, and study design and flexibility were huge (26). Evidence from real world setting of resistance exercises on skeletal muscle mass is few. Remote resistance exercise programs thus offer a series of advantages for real world use (27). Remote programs can be accessed from any location at any time, eliminating the need for transportation to a gym or fitness center. This is especially beneficial for individuals with mobility issues or those living in areas with limited access to fitness facilities. Remote exercise programs can be highly personalized to meet the specific needs and limitations of elderly individuals. This ensures that exercises are both safe and effective, contributing to improvements in strength, balance, and functional ability without risking injury. By reducing or eliminating the need for clinical visits, personal trainers, or specialized equipment, remote resistance exercises can provide a cost-effective way to combat sarcopenia. This is particularly important for those on a fixed income, such as many elderly individuals. The Joymotion®; incorporates elements of gamification, social interaction, and progress tracking, which can significantly enhance motivation and adherence. Being part of a virtual community or having the ability to share achievements with friends and family can provide additional encouragement to continue with the program. The programs and instructions within the Joymotion®; are designed based on the Standards of Care in Diabetes by the American Diabetes Association (28), and the consensus statement on exercises/physical activity in individuals with T2D from the American College of Sports Medicine (18), which provides interventions that are scientifically proven to be effective in improving strength, mobility, and frailty among elderly patients with T2D. This study with real world evidence provided a flexible, personalized, and accessible means of engaging in remote resistance exercises, representing a valuable tool in the fight against frailty, empowering individuals to maintain and improve their health and independence.

In our study, we also analyzed the potential confounding factors associated with the improvement of skeletal muscle mass. We found that elderly patients with long duration of diabetes may have a great improvement in SMI. Interestingly, high hemoglobin A1c level may also contribute to a significant improvement in SMI. Although we did not observe any association between insulin use and changes in skeletal muscle mass, insulin plays a critical role in muscle protein synthesis and glucose uptake in muscle tissues (29, 30). Therefore, individuals who manage diabetes without insulin might have a metabolic environment more conducive to gaining muscle mass when engaged in resistance training. The positive correlation between the duration of diabetes, as well as the hemoglobin A1c levels at baseline and SMI improvement might seem counterintuitive, as long-term diabetes along with poor glycemic control is often associated with complications that could impair muscle function. However, this finding could indicate that individuals with a longer history of diabetes and poorer glycemic control have more to gain from engaging in a resistance exercise program. It’s possible that these individuals had greater initial muscle loss or more pronounced muscle quality deterioration, thereby experiencing more significant gains when exposed to resistance training. Engaging in resistance exercise can lead to various metabolic adaptations that improve muscle protein synthesis, increase glucose uptake by muscles, and enhance insulin sensitivity (31, 32). These adaptations could be more pronounced in individuals who start with higher levels of metabolic dysfunction. Further studies are needed to validate these hypotheses.

The association between resistance exercises and sarcopenia is underpinned by several biological mechanisms and physiological adaptations that contribute to improved health outcomes and a reduction in frailty indicators. As aging is associated with sarcopenia, resistance training can stimulate muscle protein synthesis and inhibits protein degradation through the activation of the mammalian target of rapamycin (mTOR) pathway (33), leading to increases in muscle mass and strength. Resistance exercises also enhance neuromuscular efficiency, improving the recruitment of muscle fibers, and increasing motor unit activation. The mechanical stress applied to bones during resistance training stimulates osteogenesis (34), helping to prevent or reverse osteopenia and osteoporosis, which contributes to sarcopenia through increased fracture risk. Resistance exercises have been shown to reduce systemic inflammation (35), through the downregulation of pro-inflammatory cytokines and the enhancement of anti-inflammatory mediators. Meanwhile, AMP-activated protein kinase (AMPK) can be activated by resistance training in response to changes in cellular energy status (36). AMPK activation further promotes glucose uptake, fatty acid oxidation, and mitochondrial biogenesis by upregulating peroxisome proliferator-activated receptor gamma coactivator 1-alpha (PGC-1α) (37). Research also supports the hypothesis that resistance exercises can increase the levels of IGF-1 (38), which binds to its receptor on muscle cells, activating the Akt pathway. This also leads to increased protein synthesis and muscle cell growth, as well as decreased protein degradation. Other benefits from resistance exercises may include the reduction of myostatin and modulating the activity of NF-kB, further reducing muscle loss and dysfunction associated with sarcopenia (39).

This study underscores the potential of emerging digital technologies in managing T2D in the elderly population, along with precise quantification of skeletal muscle mass through CT imaging. However, several limitations should be fully addressed. Firstly, the study’s reliance on a self-control design may restrict the strength of the evidence provided. Secondly, it was conducted with a small participant group at a single center, suggesting the need for a larger, multi-center study to validate the findings. Lastly, the omission of dietary data, a significant confounding factor, was noted, although all participants were local residents likely sharing similar dietary habits. In future research, we plan to employ food recall questionnaires to meticulously document nutrient intake, with a focus on protein consumption, to address this limitation.

## Conclusion

Remote resistance exercises programs delivered by a smartphone application were feasible and effective in helping elderly patients with type 2 diabetes to improve their skeletal muscle mass. Future prospective randomized controlled trials are warranted to provide further evidence on this digital technology-based intervention.

## Data availability statement

The raw data supporting the conclusions of this article will be made available by the authors, without undue reservation.

## Ethics statement

The studies involving humans were approved by Institutional Review Board of the Third Affiliated Hospital of Jinzhou Medical University. The studies were conducted in accordance with the local legislation and institutional requirements. Written informed consent for participation was not required from the participants or the participants’ legal guardians/next of kin in accordance with the national legislation and institutional requirements.

## Author contributions

JY: Writing – original draft, Data curation. HT: Writing – original draft. HY: Writing – original draft, Software, Formal analysis. JL: Writing – review & editing, Data curation. YC: Writing – review & editing, Data curation. YL: Writing – review & editing. XL: Writing – review & editing. QC: Writing – review & editing. DZ: Writing – review & editing, Supervision, Methodology, Funding acquisition, Conceptualization.
